# Microbiological Quality and Resistance to an Artificial Gut Environment of Two Probiotic Formulations

**DOI:** 10.3390/foods10112781

**Published:** 2021-11-12

**Authors:** Diletta Mazzantini, Francesco Celandroni, Marco Calvigioni, Adelaide Panattoni, Roberto Labella, Emilia Ghelardi

**Affiliations:** 1 Department of Translational Research and New Technologies in Medicine and Surgery, University of Pisa, Via San Zeno 37, 56127 Pisa, Italy; diletta.mazzantini@med.unipi.it (D.M.); francesco.celandroni@dps.unipi.it (F.C.); marco.calvigioni@med.unipi.it (M.C.); a.panattoni6@studenti.unipi.it (A.P.); 2 Sanofi Consumer Health Care, Reading, Berkshire RG6 1PT, UK; Roberto.Labella@sanofi.com; 3 Research Center Nutraceuticals and Food for Health-Nutrafood, University of Pisa, 56127 Pisa, Italy

**Keywords:** probiotics, quality, viable cells, identification, gastrointestinal behavior

## Abstract

The quality control of probiotic products is the focus of numerous organizations worldwide. Several studies have highlighted the poor microbiological quality of many commercial probiotic formulations in terms of the identity of the contained microorganisms, viability, and purity, thus precluding the expected health benefits and representing a potential health risk for consumers. In this paper, we analyzed the contents of two probiotic formulations, one composed of an encapsulated mixture of lactobacilli and bifidobacteria, and one by a lyophilized yeast. The microorganisms contained in the products were quantified and identified using up-to-date methodologies, such as MALDI-TOF MS and metagenomic analysis. Moreover, as acid and bile tolerance is included among the criteria used to select probiotic microorganisms, in vitro tests were performed to evaluate the behavior of the formulations in conditions mimicking the harsh gastric environment and the intestinal fluids. Our results indicate the high quality of the formulations in terms of the enumeration and identification of the contained organisms, as well as the absence of contaminants. Moreover, both products tolerated the acidic conditions well, with encapsulation providing further protection for the microorganisms. A good tolerance to the simulated artificial intestinal conditions was also evidenced for both preparations.

## 1. Introduction

In recent years, the market for probiotics, i.e., “live microorganisms that, when administered in adequate amounts, confer health benefits on the host” [1], has dramatically increased. In parallel to studies defining the probiotic properties of microbial species and strains, the number of foods, food supplements, drugs, and medical devices containing probiotics is growing worldwide. Nevertheless, this enthusiastic market frequently encounters reports of probiotic formulations with poor microbiological quality, in terms of the identification and viability of the microorganisms contained therein [2,3,4,5,6,7,8]. These qualitative flaws may be the result of poor quality controls by manufacturers, as well as the application of methodologies by investigators that are inadequate or not rigorous enough [8]. The quality control of probiotic products is the focus of numerous organizations worldwide, with the European Society for Pediatric Gastroenterology, Hepatology, and Nutrition highlighting the importance of a more stringent control of commercialized probiotic products [9]. Thus, the qualitative and quantitative correspondence between what is stated on the label and what is contained in a formulation is a fundamental requisite that should be fulfilled by existing and new probiotic formulations. In fact, according to the Food and Agriculture Organization of the United Nations (FAO), the World Health Organization (WHO), the International Probiotics Association, and the Council for Responsible Nutrition guidelines for probiotics, product labels should indicate the minimum amount of living cells by the end of shelf life as well as the identity of each contained microbe [10,11].

The administration of probiotic preparations with an adequate amount of living organisms is crucial to obtaining the desired beneficial health effect [12]. This amount is not universally established for all products, but derives from in vitro and in vivo tests, and depends on the stability of the contained species and strains. However, some countries have established a minimum daily amount of probiotics that should be administered [13]. 

As the health benefits conferred by probiotics are generally species- and, sometimes, strain-specific [1,14], the identification of all the microbes declared to be contained in a commercial product represents a pivotal issue. The use of up-to-date identification methodologies is highly recommended, especially for multi-species and multi-strain formulations [8,15]. In particular, a combination of culture-dependent and culture-independent methods should be adopted to objectively evaluate the microbial composition of probiotic products [8]. Furthermore, the use of these methods also permits the monitoring of contaminant microorganisms (i.e., indicator microbes, pathogens, and microbes typical of the manufacturing environment), whose presence indicates significant qualitative flaws [8,16,17].

To exert their beneficial effects on the health of the host, orally administered probiotics need to pass through the harsh gastrointestinal tract unharmed and transiently colonize the intestine [9,18]. Therefore, the evaluation of acid and bile tolerance using in vitro tests is among the criteria used to select probiotic microorganisms before their inclusion in probiotic products [10,16,19]. In addition, the encapsulation of microbes is a procedure that is commonly adopted by manufacturers to protect microorganisms during their transit through the gastrointestinal tract [20,21].

In this paper, we analyzed the content, in terms of the quantification and identification of the contained microorganisms, of two probiotic formulations sold in different countries with different trade names consisting of an encapsulated mixture of lactobacilli and bifidobacterial, and a lyophilized yeast, respectively. Moreover, the survival in artificial gastric juices and the behavior in artificial intestinal juice of the microorganisms contained in the two formulations were evaluated.

## 2. Materials and Methods

### 2.1. Probiotic Products

The dietary supplements Microbiosys Confort Digestif, i.e., Microbiosys (batch number: 3039, expiration date: 09/2021, Sanofi, Paris, France; also referred to as Buscobiota in the UK and Ireland, Normabiotic Complete in Poland, and Bioflorin Daily Balance in Switzerland), and Enterogermina Viaggi (batch number: C06406, expiration date: 06/2021, Sanofi, Paris, France; also referred to as DioraByota in the UK and Ireland, and Normabiotic Podróż in Poland) were used in this study. All formulations were purchased in pharmacies by the investigators and analyzed before the expiration date. The study was performed in 2020.

### 2.2. Quantification of Living Microbes

The Microbiosys capsules and the Enterogermina Viaggi sachets were opened and their contents dissolved in 10 mL of sterile PBS (1 M KH_2_PO_4_, 1 M K_2_HPO_4_, 5 M NaCl, pH 7.2) before the analyses were performed. The suspensions were serially diluted and seeded on different media in order to selectively differentiate the species contained in the formulations. Aliquots (100 μL) of Microbiosys were seeded on Tryptone Soy Agar (TSA; Thermo Fisher Scientific, Waltham, MA, USA) to allow for the growth of all the microbes contained in the formulation. For the selective isolation of *Bifidobacterium* spp. declared to be contained in the product, the aliquots were also seeded on Bifidus Selective Medium (BSM; Sigma-Aldrich, Saint Louis, MO, USA) containing 0.116 g/L BSM supplement (Sigma-Aldrich, Saint Louis, MO, USA). TSA and BSM plates were incubated for 72 h at 37 °C in anaerobic atmosphere using Oxoid^TM^ AnaeroGen^TM^ (Thermo Fisher Scientific, Waltham, MA, USA). Aliquots (100 μL) of the Enterogermina Viaggi suspension were seeded on YPD (20 g/L bacteriological peptone, 10 g/L yeast extract, and 20 g/L glucose) agar plates and incubated at 30 °C for 48–72 h. The number of colony forming units (CFUs) was determined and the total amount of CFUs contained in one dose of each formulation (one capsule and one sachet) was calculated. 

### 2.3. Culture-Dependent Identification of Probiotic Microbes by Matrix-Assisted Laser Desorption/Ionization Time-of-Flight Mass Spectrometry (MALDI-TOF MS) 

For each product, the colonies grown on solid media were carefully examined for morphology and all phenotypically different colonies were subjected to identification by MALDI-TOF MS in a MALDI Biotyper Microflex LT mass spectrometer (Bruker Daltonik, Bremen, Germany). When a colony was directly spotted on the MALDI plate, it was overlaid with 1 μL of saturated formic acid, and air-dried. Subsequently, 1 μL of acetonitrile-matrix solution was added to each spot, and air-dried. The loaded plate was placed in the instrument according to the manufacturer’s instructions. The mass spectra were acquired, imported into the integrated MALDI Biotyper software (version 3.0), and analyzed using standard pattern matching with a default setting. A score of ≥2.00 indicated identification at the species level, while a score from 1.70 to 1.99 indicated identification at the genus level. Any score under 1.70 meant no significant similarity of the obtained spectrum with any database entry. 

### 2.4. Culture-Independent Identification of Probiotic Microbes by Genomic DNA Extraction and Metagenomic Analysis

Genomic DNA was extracted from the whole Microbiosys formulation using the QIAamp DNA Mini kit (QIAGEN, Hilden, Germany) following the manufacturer’s instructions. To perform genomic DNA extraction from the whole Enterogermina Viaggi formulation, an adapted protocol was applied [22]. Briefly, Enterogermina Viaggi content was dissolved in 10 mL of sterile water and cells were harvested by centrifuging at 4500 rpm for 10 min at 4 °C. Cell lysis was performed by vigorously vortexing for 3 min with 0.3 g of glass beads (from 0.45 to 0.52 mm diameter; Merck KGaA, Darmstadt, Germany) in 0.2 mL of lysis buffer (2% TritonX-100, 1% SDS, 100 mM NaCl, 10 mM Tris-HCl, 1 mM EDTA, pH 8.0) and 0.2 mL of 1:1 phenol-chloroform (Merck KGaA, Darmstadt, Germany). After vortexing, 0.2 mL of TE (10 mM Tris-HCl, 1 mM EDTA, pH 8.0) was added to the lysate. The mixture was centrifuged at 4500 rpm for 10 min and the aqueous phase was transferred to new tubes. An amount of 10 mg/mL of RNAse (Merck KGaA, Darmstadt, Germany) was added and the mixture was incubated at 37 °C for 60 min. An amount of 0.5 mL of 25:24:1 phenol-chloroform-isoamyl alcohol solution (Merck KGaA) was added and the tubes were centrifuged at 14,000 rpm for 5 min. The aqueous phase was transferred to new tubes and 0.5 mL of 24:1 chloroform-isoamyl alcohol solution (Merck KGaA, Darmstadt, Germany) was added. After centrifugation at 14,000 rpm for 10 min, the aqueous phase was transferred to new tubes and 0.6 volumes of isopropanol (Merck KGaA, Darmstadt, Germany) was added. The tubes were centrifuged at 14,000 rpm for 10 min and the pellets were washed with 70% ethanol (Merck KGaA, Darmstadt, Germany). After centrifugation at 14,000 rpm for 10 min, the extracted DNA was suspended in 30 µL of sterile water. Genomic DNA extracted from the Microbiosys and Enterogermina Viaggi formulations were subjected to metagenomic analysis of bacterial 16S rDNA and eukaryotic 18S rDNA. Both sequencing and data analysis were carried out by Novogene (UK Company Limited). Briefly, the V4, V3–V4, and V4–V5 regions of the 16S rRNA gene, and the V4 region of the 18S rRNA gene were amplified using the Phusion^®^ High-Fidelity PCR Master Mix (New England BioLabs, Ipswich, MA, USA), and the PCR products were purified with the QIAGEN Gel Extraction Kit (QIAGEN, Hilden, Germany). Libraries were generated with the NEBNext^®^ UltraTM DNA Library Prep Kit for Illumina and quantified via Qubit and qPCR. Amplicon sequencing was carried out on the HiSeq Illumina platform (Illumina, San Diego, CA, USA). Sequence analysis was performed using Uparse software (Uparse version 7.0.1001). Regarding the 16S rRNA coding regions, Mothur software was run against the SSU-rRNA database of the SILVA database (http://www.arbsilva.de/ (accessed on 1 September 2021) to obtain the annotations regarding the taxonomic ranks (i.e., kingdom, phylum, class, order, family, genus, species). The RDP Classifier (version 2.2) and the Silva database were used for the 18S rRNA gene sequences.

### 2.5. Resistance of Probiotic Microbes to Artificial Gastric Juices

Microbes contained in Microbiosys and Enterogermina Viaggi were tested for resistance in two artificial gastric juices (i.e., ASTM and USP), both having an initial pH of 1.5 and 3.0. The ASTM juice consisted of a solution of 0.07 N hydrochloric acid with an initial pH of 1.5 or 3.0 at 37 °C, as specified by the American Society of Testing Materials [23]. The USP juice consisted of 0.03 M sodium chloride, 0.084 M hydrochloric acid, and 0.32% pepsin with an initial pH of 1.5 or 3.0 at 37 °C, as recommended by the U.S. Pharmacopoeia [24]. The contents of the Microbiosys capsules and the Enterogermina Viaggi sachets were directly dissolved in 5 mL of artificial gastric juices and the total amount of CFUs inoculated in the fluids (i.e., 0 min) was determined by plate counting. The samples were incubated at 37 °C for 30, 60, and 120 min. At each time point, aliquots (100 μL) of the suspensions were seeded on solid media (i.e., TSA for Microbiosys and YPD for Enterogermina Viaggi) to determine the total CFUs contained in one dose of product. Microbial survival at the end of incubation (i.e., 120 min) was calculated according to the following equation [25]:

$$\mathrm{Survival}\;\mathrm{rate}\;\left(\%\right)=\frac{\log\mathrm{CFUs}\;\mathrm{of}\;\mathrm{viable}\;\mathrm{cells}\;\mathrm{survived}}{\log\mathrm{CFUs}\;\mathrm{of}\;\mathrm{initial}\;\mathrm{viable}\;\mathrm{cells}\;\mathrm{inoculated}}\times 100$$

### 2.6. Resistance of Probiotic Microbes in Simulated Intestinal Fluid

Microbes contained in Microbiosys and Enterogermina Viaggi were tested for tolerance to simulated intestinal conditions characterized by an alkaline pH [26,27]. The contents of the Microbiosys capsules and the Enterogermina Viaggi sachets were directly dissolved in 5 mL of artificial intestinal fluid (0.1% pancreatin, 0.3% Oxgall bile salts, pH 8.0) and the total amount of inoculated CFUs (i.e., 0 min) was determined by plating the aliquots (100 μL) on solid media. The suspensions were incubated for 30, 60, 120, 240, and 360 min at 37 °C. At each time point, the total CFUs contained in one dose of product were determined using the plate count method. The microbial survival at the end of incubation (i.e., 360 min) was calculated as described above [25].

### 2.7. Behavior of Microbiosys Intact Capsules in Simulated Gastric and Intestinal Conditions

To evaluate the behavior of the Microbiosys capsules in simulated gastric conditions, intact capsules were immersed in 5 mL of ASTM (pH 1.5 and 3.0) and USP (pH 1.5 and 3.0) juices and incubated for 30, 60, and 120 min at 37 °C. At each time point, the total CFUs released by the capsules were determined using the plate count method. As models of intact capsules that usually reach the intestine, the Microbiosys capsules were suspended in 5 mL of artificial intestinal fluid prepared as described above and incubated at 37 °C for up to 6 h. At 30, 60, 120, 240, and 360 min, the total content of microbes released by the capsules was determined by plating aliquots (100 μL) of the suspensions on solid media. For all assays, the total amount of CFUs determined in the contents of the Microbiosys capsules was considered as inoculum at 0 min. At each time point, the release rate of microbes by the capsules was calculated as follows:

$$\mathrm{Release}\;\mathrm{rate}\;\left(\%\right)=\frac{\log\mathrm{CFUs}\;\mathrm{of}\;\mathrm{viable}\;\mathrm{cells}\;\mathrm{released}\;\mathrm{by}\;\mathrm{the}\;\mathrm{capsules}}{\log\mathrm{CFUs}\;\mathrm{of}\;\mathrm{viable}\;\mathrm{cells}\;\mathrm{inoculated}\;}\times 100$$

### 2.8. Statistical Analysis

DNA extraction from the formulations was performed three times on separate days. The other experiments were repeated four times on separate days and, for each replicate, plating was performed in triplicate. Data were expressed as the mean ± standard deviation (S.D.). Both statistical analyses and graphs were realized on GraphPad Prism version 8.0.2 (GraphPad Software Inc., San Diego, CA, USA). For assessing the microbial viability and capsule release in the simulated gastric juices, a one-way analysis of variance (ANOVA) for repeated measures followed by Sidak’s multiple comparisons test were applied to compare the total CFUs obtained at each time point with the total CFUs inoculated in the juices (i.e., 0 min). For evaluating microbial viability and capsule release in simulated intestinal conditions, ANOVA for repeated measures followed by Tukey’s HSD test were used to compare the total CFU amounts obtained at each time point. A two-sided *p*-value (*p*) < 0.05 was considered significant.

## 3. Results

### 3.1. Quantification and Identification of the Microbes Contained in the Commercial Formulations 

Considering the importance of quality controls for commercialized probiotic products, we analyzed the content of the two formulations in terms of the quantification and identification of the contained microorganisms. Table 1 reports the amount of CFUs declared by the manufacturers on the products labels (i.e., claimed CFUs) and the mean number of CFUs experimentally obtained (i.e., total CFUs). Both CFU counts refer to the content of the daily dose of the original product (one capsule and one sachet). Microbiosys and Enterogermina Viaggi were found to be compliant with the label claims for the content of living microbes, as the determined total CFU amount was found to be higher than that labelled by the manufacturers.

Both culture-dependent and culture-independent methods were applied to decipher the microbial composition of the formulations. All morphologically different colonies isolated from each product were subjected to identification by MALDI-TOF MS. In parallel, genomic DNA was extracted from the whole formulations and subjected to metagenomic analysis. Table 2 reports the results of both identification procedures.

By combining both identification methods, a total of four species were detected in Microbiosys (Table 2). Possibly due to the low number of spectra contained in the MALDI-TOF MS database for *Bifidobacterium* spp., we were unable to identify the two *Bifidobacterium* species present in Microbiosys using this technique. On the other hand, these species were identified by 16S rDNA sequencing. *Bifidobacterium animalis* spp. *lactis* was identified as *Bifidobacterium animalis* on the basis of the sequence of 16S rDNA [28]. *Lactobacillus helveticus* was correctly identified by MALDI-TOF MS, while it was identified as *Lactobacillus gallinarum* using metagenomic sequencing. As *L. helveticus* and *L. gallinarum* are closely related species and show an almost identical 16S rRNA gene sequence [29], they were indistinguishable by sequencing of this gene. No eukaryotic microorganisms were identified in Microbiosys by 18S rDNA sequencing (data not shown). 

The declared *Saccharomyces cerevisiae* var. *boulardii* was correctly identified as *Saccharomyces cerevisiae* by MALDI-TOF MS, as reported in the literature for such a species [30]. Using 18S rDNA sequencing, this species was identified as *Saccharomyces cariocanus*. This result was expected as this *Saccharomyces* species show a 99.9% similarity in their 18S rDNA sequence, thus being indistinguishable in 18S rDNA analysis [31]. 

Overall, our findings indicate that Microbiosys and Enterogermina Viaggi contain the species declared by the manufacturers on the product label and are microbiologically pure.

### 3.2. Survival of Microbes Contained in Microbiosys and in Enterogermina Viaggi in Simulated Gastric Juices

**Figure 1:**
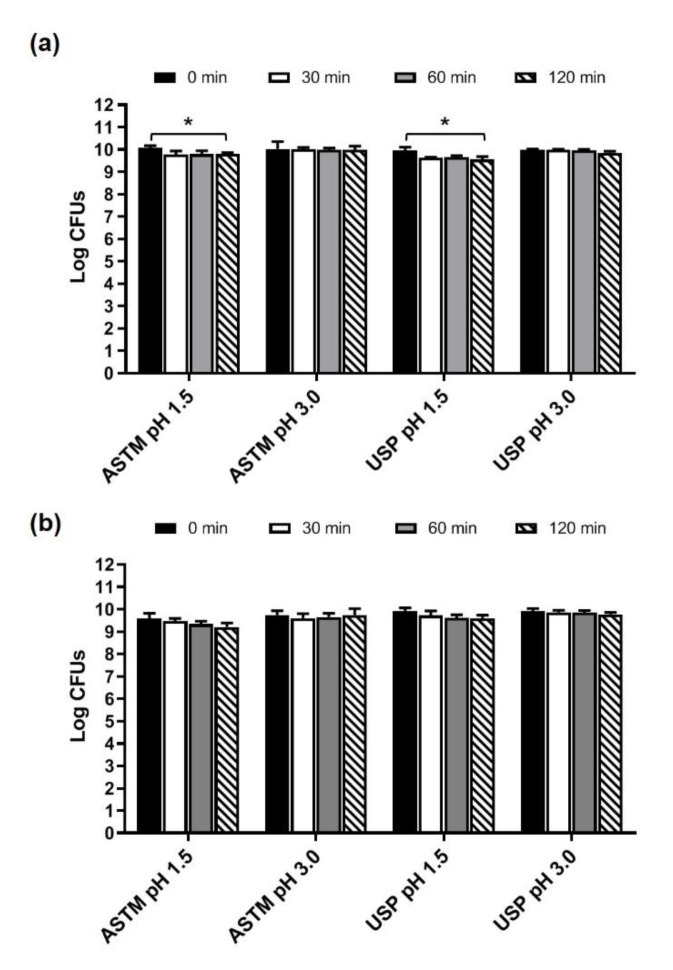
Survival of microorganisms contained in Microbiosys (**a**) and Enterogermina Viaggi (**b**) in the ASTM- and USP-simulated gastric fluids at pH 1.5 and 3.0. Microbial counts were carried out at 0, 30, 60, and 120 min of incubation and expressed as Log CFUs. * *p* < 0.05.

Microbiosys capsules and Enterogermina Viaggi sachets were opened and their contents dissolved in the ASTM- and USP-simulated gastric juices, both at pH 1.5 and 3.0. The juices were incubated at 37 °C and the total amount of CFUs for a dose of product was determined by plate counting at 0, 30, 60, and 120 min of incubation. As shown in Figure 1a, the total amount of microbes contained in Microbiosys remained stable for up to 60 min in the ASTM and USP juices at pH 1.5. A slight decrease in living bacteria was registered after 120 min of incubation in both juices (*p* < 0.05 compared to 0 min). Nevertheless, the survival rates at 120 min in the ASTM and USP gastric juices (pH 1.5) were 97.41% and 95.97%, respectively, thus indicating that a substantial number of bacteria survive in the artificial fluids for up to 120 min. When the microbes contained in Microbiosys were tested for the ability to survive in the ASTM and USP juices at pH 3.0, no differences in the amount of CFUs over time were found (Figure 1a). In fact, the survival rates calculated after 120 min of incubation were 99.59% and 98.68% in the ASTM and USP juices (pH 3.0), respectively.

As shown in Figure 1b, the *S. cerevisiae* strain present in the Enterogermina Viaggi formulation was found to be highly tolerant to acidity, as no decrease in yeast viability was recorded after 120 min of incubation in all simulated gastric juices. At this time, the calculated survival rates in ASTM at pH 1.5, ASTM at pH 3.0, USP at pH 1.5, and USP at pH 3.0 were 96.15%, 99.99%, 96.76%, and 98.59%, respectively. 

Taken together, these results indicate that microbes contained in Microbiosys and Enterogermina Viaggi show a good tolerance to the harsh gastric simulated conditions.

### 3.3. Behavior of Microbes Contained in Microbiosys and in Enterogermina Viaggi in Simulated Intestinal Fluid

To evaluate the behavior of the microbes contained in Microbiosys and Enterogermina Viaggi in simulated intestinal conditions, Microbiosys capsules and Enterogermina Viaggi sachets were opened and their contents dissolved in the simulated intestinal fluid at pH 8.0. The juice was incubated at 37 °C and the total amount of CFUs for a dose of product was determined by seeding aliquots on solid media at 0, 30, 60, 120, 240, and 360 min of incubation.

**Figure 2:**
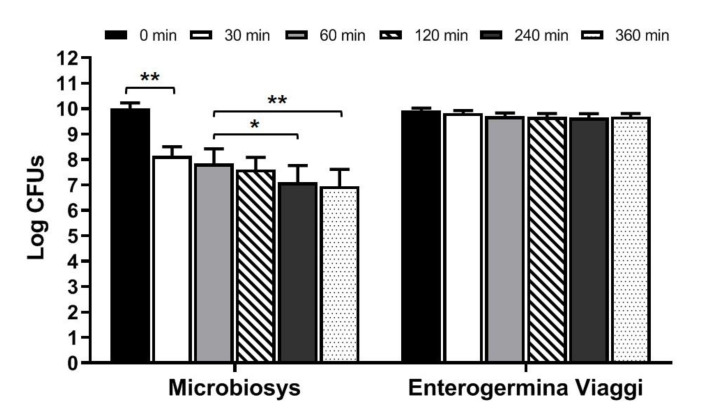
Behavior of microbes contained in Microbiosys and Enterogermina Viaggi in simulated intestinal juice (pH 8.0). Microbial counts were carried out at 0, 30, 60, 120, 240, and 360 min, and expressed as Log CFUs. * *p* < 0.05, ** *p* < 0.01.

As shown in Figure 2, a significant reduction in cell viability was observed for the bacteria contained in Microbiosys starting from 30 min of incubation (*p* < 0.01 compared to 0 min). The total amount of living microbes decreased again after 240 and 360 min of incubation (*p* < 0.05 and *p* < 0.01 compared to 60 min, respectively). Nevertheless, the survival rate determined at 360 min was 69.40%, indicating that most microbes survived in the simulated intestinal fluid for a prolonged period.

Yeasts contained in Enterogermina Viaggi survived well in the simulated fluid for up to 360 min (Figure 2). In fact, no variations in the total amount of CFUs over time was recorded, and the survival rate calculated at 6 h was 97.52%. 

Overall, our findings indicate that microbes contained in both formulations show good tolerance to simulated intestinal conditions.

### 3.4. Behavior of Microbiosys Intact Capsules in Simulated Gastrointestinal Conditions

As Microbiosys is administered to humans as capsules, we speculated about whether the capsules could protect the contained microbes during transit through the gastrointestinal tract. 

To test the capsule resistance in simulated gastric conditions, intact Microbiosys capsules were directly immersed into ASTM and USP gastric juices at pH 1.5 and 3.0. The dissolution of the capsules was monitored, and the amount of microbes released in the juices was determined by plating aliquots of the suspension on TSA after 30, 60, and 120 min of incubation. The amount of bacteria quantified in the contents of the Microbiosys capsules was considered as inoculum. Table 3 indicates the Log CFUs released in the ASTM and USP juices (pH 1.5 and 3.0) by the capsules and the calculated release rates.

As shown in Table 3, the capsules did not completely dissolve during incubation in all simulated gastric juices for up 120 min. In fact, for all fluids, the total amount of bacteria released by the capsules was significantly lower at each time point compared to the inoculum (*p* < 0.05). These results highlight the protection that the capsules conferred to the contained microbes from the acidic conditions of the artificial gastric fluids. 

As models of capsules that usually reach the intestine intact, the Microbiosys capsules were tested for their ability to protect bacteria in simulated intestinal conditions. Intact capsules were incubated in the simulated intestinal fluid (pH 8.0) for up to 360 min and visually monitored to evaluate their dissolution. The total amount of bacteria released by the capsules was determined after 30, 60, 120, 240, and 360 min of incubation using the plate count method. Table 4 indicates the Log CFUs released in the simulated intestinal fluid (pH 8.0) and the calculated release rates. 

From visual inspection, the Microbiosys capsules appeared to be intact for up to 120 min of incubation in the simulated intestinal fluid, while they were completely dissolved at 240 min. Due to the presence of almost intact capsules after 30 min of incubation, the amount of microbes released by the capsules at this time point was significantly lower compared to the inoculum (*p* < 0.01, Table 4). The number of viable cells progressively increased until 240 min of incubation as a consequence of capsule dissolution (*p* < 0.01 compared to 30 min). The amount of microbes quantified after 240 and 360 min of incubation was significantly lower compared with the inoculum (*p* < 0.01). Nevertheless, the finding that 78.78% of microbes were still vital after 360 min of incubation indicates that most microbes survive in the intestinal juice for up to 360 min (Table 4).

Taken together, these findings indicate that the encapsulation of Microbiosys bacteria by the manufacturers is effective in protecting the microbes from harsh gastrointestinal conditions.

## 4. Discussion

To confer beneficial health effects, commercial probiotic products should fulfill some basic quality requirements as indicated in the existing guidelines for probiotics [10,11]. Nevertheless, the analysis of some commercial products revealed many discrepancies between the label claims and the effective microbial content, thus potentially precluding any beneficial effect of these formulations [2,3,4,5,6,7,8,15].

In this study, the amount of microorganisms contained in Microbiosys and Enterogermina Viaggi was determined using the plate count method. Although several culture-independent techniques for microbial quantification in probiotic formulations have been alternatively proposed [32,33,34], cultivability is considered to be the main feature of viable cells, and the plate count method is still applied as the gold-standard in industrial controls [16,35,36]. As result, we found that both formulations contained a slightly higher total amount of living microbes than that stated on the label by the manufacturers. This finding was expected as the product analyses were performed many months before expiry. In fact, as manufacturing, packaging, and some environmental factors are known to affect microbial viability in commercial formulations, manufacturers often include overage amounts of microorganisms to guarantee the stated amount of microbes until product expiry [37,38].

The identification of the microbes contained in probiotic formulations can represent a major challenge, particularly for multi-species products. In this study, the microorganisms of Microbiosys and Enterogermina Viaggi were identified using both culture-dependent MALDI-TOF MS and culture-independent metagenomic sequencing to gain objective data on their microbial make-up. In fact, while culture-dependent techniques are strictly linked to microbial cultivability in the experimental conditions used and to the ability of investigators to morphologically discriminate colonies, culture-independent methods detect all microbes contained in the formulations independently from their viability [8,14]. MALDI-TOF MS and metagenomic sequencing have successfully been used for the identification of probiotics in many different studies [3,4,5,7,27,39,40,41,42,43,44]. 

In this study, MALDI-TOF MS correctly identified both *Lactobacillus* species of Microbiosys, while it failed to detect *B. animalis* and *B. bifidum*, although some *Bifidobacterium*-presumptive colonies were obtained on BSM plates. However, both *Bifidobacterium* species were detected by 16S rRNA gene sequencing, thus indicating that these species were effectively contained in the formulation. These results correlate with the evidence that MALDI-TOF MS has been shown to fail in identifying some species when the number of species- and strain-specific spectra included in the MALDI database is low [45]. A previous study suggested that the development of a dedicated in-house library containing MALDI TOF MS spectra of various reference strains could improve the identification accuracy [46]. Metagenomic sequencing misidentified *L. helveticus* in Microbiosys. Similarly, while *S. boulardii* in Enterogermina Viaggi was correctly identified by MALDI-TOF MS, it was misidentified by 18S rDNA gene sequencing. These apparently contrasting results could be due to technical differences in the applied methodologies. In fact, sequencing identifies microbes at the base of the sequence of short portions of the 16S rRNA and 18S rRNA genes that can be shared by closely related species, thus resulting in the inability to discriminate these species [47]. In stark contrast, MALDI-TOF MS identification is performed by comparing peptide mass spectra generated for many abundant cytosolic proteins, thus the results are more discriminative in some cases [48]. Lastly, no additional microbes other than those declared by the manufacturers were detected in Microbiosys or Enterogermina Viaggi using either identification method, thus indicating that the formulations were microbiologically pure. Our findings suggest that the use of two methods should be adopted for correctly identifying microbes in commercial products. In fact, in a previous study analyzing the quality of dietary supplements sold in Slovenia, MALDI-TOF MS alone was not accurate in distinguishing some closely related *Lactobacillus* species. In these cases, the correct identification was obtained by pairing MALDI-TOF MS with species-specific PCR [46].

The use of artificial fluids simulating the gastrointestinal conditions is a commonly adopted method to evaluate the potential behavior of probiotic microbes during their transit through the gastrointestinal tract [26,27]. In this study, *S. boulardii* in Enterogermina Viaggi was found to highly tolerate the harsh conditions of the ASTM- and USP-simulated gastric juices, as well as those of the artificial intestinal fluid, for the whole period tested. These results are in accordance with the high acid and bile salt resistance documented for this species in previous studies [27,49,50,51]. In fact, in previous studies, *S. boulardii* contained in Codex (Zambon, Bresso, Italy) was also shown to be able to grow in simulated gastrointestinal conditions [27,51].

The bacteria in Microbiosys were found to survive in very large amounts in all the simulated gastric juices for prolonged periods, although a slight reduction in their number was observed in the ASTM and USP gastric juices at pH 1.5 after 120 min of incubation. Interestingly, the assays conducted using intact Microbiosys capsules indicated that capsules do not dissolve in simulated gastric juices for up to 120 min, and this may completely preserve the viability of the contained microbes. When the Microbiosys bacteria were tested for their tolerance in the artificial intestinal fluid, a significant reduction in their number was observed from 30 min of incubation onwards. This result was expected as bile resistance in the *Lactobacillus* and *Bifidobacterium* genera represents a species- and strain-specific feature [52]. However, the finding that approximately 69% of the microbes were found to be alive after 360 min of incubation in the juice indicates a good resistance to the simulated intestinal conditions. Interestingly, when the intact Microbiosys capsules were tested in the artificial intestinal fluid, approximately 79% of the bacteria were found to be released by the capsules after 6 h of incubation, indicating that encapsulation guarantees that a large amount of microbes potentially reach the gut. No studies investigating identical strain mixtures are available in the literature, and reports describing potential gastrointestinal behavior are mainly restricted to mono-strain products containing *L. rhamnosus* GG. This organism, analyzed from Dicoflor (Dicofarm, Rome, Italy), was found to suffer extreme acidity and the presence of bile, thus showing poor tolerance to the simulated gastrointestinal conditions [27,51]. In contrast, in a sophisticated study investigating the resistance properties of several probiotic *Bifidobacterium* strains, *B. animalis* subsp. *lactis* LAFTI B94 was found to tolerate both acids and bile very well [53].

## 5. Conclusions

The results of this study indicate the high quality of Microbiosys and Enterogermina Viaggi in terms of the enumeration and identification of the contained organisms. In fact, the number of microbes contained in the products was found to be slightly higher than that declared by the manufacturers. Moreover, the microorganisms contained in both formulations were able to survive in conditions mimicking the gastrointestinal tract. The slightly lower survival observed for the bacteria contained in Microbiosys could be strengthened by the inclusion of the microorganisms in a capsule, which would guarantee an appropriate grade of protection for the bacteria against acidic conditions and bile salts. In conclusion, it is advisable that any new or existing probiotic formulation is qualitatively and quantitatively analyzed using up-to-date methodologies to obtain a reliable overview of the product quality. In addition, the evaluation of the acid and bile tolerance of microbes contained in oral probiotics should be mandatory to guarantee the ability of these organisms to reach the intestine alive and carry out their beneficial activities. We hope that the present study can serve as an example for designing future studies focusing on the quality of probiotic products. 

## Figures and Tables

**Table 1:** Quantification of the microbes contained the formulations (per dose).

Formulation	Form	Claimed CFUs	Total CFUs (Mean ± S.D.)
Microbiosys	Capsule	5 × 10^9^	2.86 ± 2.28 × 10^10^
Enterogermina Viaggi	Sachet	6 × 10^9^	1.22 ± 0.82 × 10^10^

**Table 2:** Identification of the microbes contained in the formulations.

Product	Claimed Species	MALDI-TOF	Metagenomic
Microbiosys	*Lactobacillus rhamnosus* Rosell^®^-11 ^1^ *Lactobacillus rhamnosus* GG ^1^	*L. rhamnosus*	*L. rhamnosus*
	*Lactobacillus helveticus* Rosell^®^-52	*L. helveticus*	*L.gallinarum*
	*Bifidobacterium animalis* subsp. *lactis* LAFTI^®^ B94	N.I. ^2^	*B. animalis*
	*Bifidobacterium bifidum* HA-132	N.I. ^2^	*B. bifidum*
Enterogermina Viaggi	*Saccharomyces boulardii*	*S. cerevisiae*	*S. cariocanus*

^1.^ Now *Lacticaseibacillus rhamnosus* (indicated as *L. rhamnosus* throughout the paper), according to the new nomenclature for this species. ^2.^ Not identified.

**Table 3:** Behavior of Microbiosys intact capsules in ASTM- and USP-simulated gastric juices (pH 1.5 and 3.0).

Juice	Inoculum	30 Min	60 Min	120 Min
ASTM pH 1.5	10.330 ± 0.304 ^1^	9.190 ± 0.117 ^1^ 88.97 ^2^	8.890 ± 0.288 ^1^ 86.07 ^2^	9.293 ± 0.223 ^1^ 88.27 ^2^
ASTM pH 3.0	10.330 ± 0.304 ^1^	8.540 ± 0.368 ^1^ 82.68 ^2^	8.974 ± 0.267 ^1^ 86.87 ^2^	9.295 ± 0.095 ^1^ 89.98 ^2^
USP pH 1.5	10.330 ± 0.304 ^1^	8.287 ± 0.360 ^1^ 80.67 ^2^	8.463 ± 0.391 ^1^ 83.84 ^2^	9.102 ± 0.257 ^1^ 89.78 ^2^
USP pH 3.0	10.330 ± 0.304 ^1^	8.204 ± 0.698 ^1^ 79.42 ^2^	9.192 ± 0.122 ^1^ 88.98 ^2^	9.423 ± 0.08 ^1^ 91.22 ^2^

^1^ Log CFUs (Mean ± S.D.). ^2^ Release rate (%).

**Table 4:** Behavior of Microbiosys capsules in the simulated intestinal juice (pH 8.0).

	Log CFUs ^1^	Release Rate (%)
Inoculum	10.330 ± 0.304	N.A. ^2^
30 min	7.367 ± 0.156	71.32
60 min	8.110 ± 0.671	78.51
120 min	8.280 ± 0.144	80.16
240 min	8.645 ± 0.145	83.70
360 min	8.137 ± 0.121	78.78

^1^ Mean ± S.D. ^2^ Not attributable.

## Data Availability

Data are available upon request to the corresponding author.
